# Translational Role of Rodent Models to Study Ventilator-Induced Lung Injury

**Published:** 2021-11-27

**Authors:** Koichi Yuki, Sophia Koutsogiannaki

**Affiliations:** 1 Cardiac Anesthesia Division, Department of Anesthesiology, Critical Care and Pain Medicine, Boston Children’s Hospital, USA; 2 Department of Anaesthesia, Harvard Medical School, USA

## Abstract

Mechanical ventilation is an important part of medical care in intensive care units and operating rooms to support respiration. While it is a critical component of medical care, it is well known that mechanical ventilation itself can be injurious to the lungs. Despite a large number of clinical and preclinical studies that have been done so far, there still exists a gap of knowledge regarding how to ventilate patients mechanically without increasing lung injury. Here, we will review what we have learned so far from preclinical and clinical studies and consider how to use preclinical models of ventilation-induced lung injury that better recapitulate the clinical scenarios.

## Introduction

Mechanical ventilation (MV) is a critical component of clinical care. MV is frequently utilized in intensive care units (ICUs) for patients with respiratory insufficiency. Acute respiratory distress syndrome (ARDS) is a severe form of lung injury and seen in approximately 10% of patients in ICUs [1]. 35% of all patients in ICUs worldwide receive MV even if they do not have ARDS [2]. MV is also used as a part of general anesthesia for surgical procedures in patients with normal lung function. 230 million patients per year require MV for major surgery [3]. While MV is supposedly administered for medical benefit to patients, it was recognized soon after its use that it could also cause structural lung damage (ventilator-induced lung injury; VILI) [4]. Thus, mitigating the chance of developing VILI is critical.

While clinical outcome studies are a must to understand existing issues, direct inspection of the lungs in patients undergoing MV to determine the nature and the extent of lung injury is almost impossible and not practical. The current diagnosis of lung injury is based on the clinical criteria such as the ratio of arterial oxygen partial pressure (PaO_2_ in mmHg) to fractional inspired oxygen (FiO_2_ expressed as a fraction) and imaging studies [5]. As a result, there are significant limitations in understanding the underlying mechanism of VILI development from clinical data. In addition, patient medical history and clinical scenarios for MV requirement are extremely diverse. In contrast, preclinical VILI models are advantageous in dissecting the mechanism of VILI development; Experimental conditions can be strictly controlled, and tissue samples are easily obtainable. A host of species has been used for preclinical studies. However, the lung anatomy, the mechanics and the immune system are not exactly the same among different species and therefore, no particular species/strategy combination developed to date can be described as a gold standard [6]. Among them, rodents are most popularly used. Thus, here we will review the role of preclinical rodent models in MV-induced lung injury research, particularly using mice.

## Differences between Mouse and Human Lungs

### Anatomical differences

The mouse lung is quite different from human lung from a structural standpoint [7]. The total lung capacity (TLC) of the mouse is about 1 mL. For human adult, it is about 5,000–6,000 mL. Mouse lung has four right lobes and one left lobe, while human lung has three right lobes and two left lobes. Mouse lung has fewer respiratory bronchioles and airway generations (13–17 generations) compared to human lung (17–21 generations). The parenchyma of the mouse lung occupies a bigger fraction of the total lung (18%) than that of the human (12%). The alveoli of the mouse lung are smaller (80 μm mean linear intercept (MFI)) than those of the human (210 μm). The blood-air barrier thickness in the mouse is smaller (0.32 μm) compared to that of the human (0.62 μm). The anatomical differences between mouse and human lungs are summarized in Table 1.

### Physiological differences

Physiological values for mouse respiratory parameters in the literature are rather divergent, possibly because it can be technically challenging to measure these variables and there may be a large inter-strain variability and/or a difference in experimental conditions [8]. Regarding adult mice breathing spontaneously, respiratory rates range from 180–350 ml/min, tidal volume (TV) from 100 μL to 200 μL (or 3–10 mL/kg), and an inspiratory fraction (inspiratory to total time of the respiratory cycle, Ti/Tt) from 0.30 to 0.35 [8,9]. A mouse at rest can use up to 3.5 mL of oxygen/gram/hour, while a human can use 0.25 mL of oxygen/gram/hour [10]. Due to this metabolic rate, respiratory rate of a mouse is quite higher than that of human, but tidal volume can be comparable. In terms of gas exchange, PaCO_2_ is much lower in mice (20–35 mmHg) compared to that in human (35–45 mmHg) [8].

### Immunological differences

Immunological responses are critical for the development and resolution of lung injury. Thus, species-specific differences should be well appreciated before designing lung injury studies. Infection is one of the major causes that lead into MV support for respiration. Professional phagocytes are the major immune cells critical for microbial clearance. Thus, understanding the difference in phagocyte characteristics among different species may be important. Major professional phagocytes include neutrophils, monocytes, macrophages and dendritic cells. Intravascular macrophages directly face the circulation for microbial ingestion. In many species, which include rodents and human, intravascular macrophages are restricted to the spleen and liver [11]. In livestock species such as sheep and pigs, however, the lungs contain pulmonary intravascular macrophages, a resident population of mature macrophages that adhere to endothelial cells in pulmonary capillaries in addition to alveolar macrophages that usually exist in most species. How these anatomical differences in immune cell profiles among species affect microbial protection is not known, but knowing these differences and potentially delineating their contribution, is an important consideration when we test two-hit models where the first hit is triggered by microbial infection.

From genomic standpoint, mouse genome is quite similar to human. The genome sequence project of mouse on the C57/BL6 background indicates that its genome has actually 99% similarity to the human genome [12]. Although there was a study raising concerns that genomic responses in mouse models poorly mimic human inflammatory diseases [13], a follow-up study using the same database suggested that genomic responses in mouse models greatly mimic human inflammatory diseases [14]. However, it is certain that there are some immunological differences between mice and humans. For example, differences have been found in Toll-like receptors (TLRs), which are one of the pattern recognition receptors for microbial products and endogenous danger signals. Specifically, TLR4 from humans and mice has been found to recognize different lipopolysaccharide (LPS) structures [15]. In addition, intra-species differences in the immunological responses are important and should be taken into consideration to human-mice differences. C57/BL6 is one of the major strains that have been used in immunology research, but it is also important to know that are differences in the immunological responses among different mouse strains when testing VILI in different strains. For instance, and as described in more details below, A/J strain is more sensitive to VILI compared to C57/BL6. As mentioned above, TLR4 pathway is described to be important in VILI, and the intra-species difference in TLR4 signaling pathway may need to be given another consideration regarding immunological responses [16].

## Current Evidence of VILI in Clinical Studies

### Ventilator-induced lung injury in ICU patients

**Figure 1:**
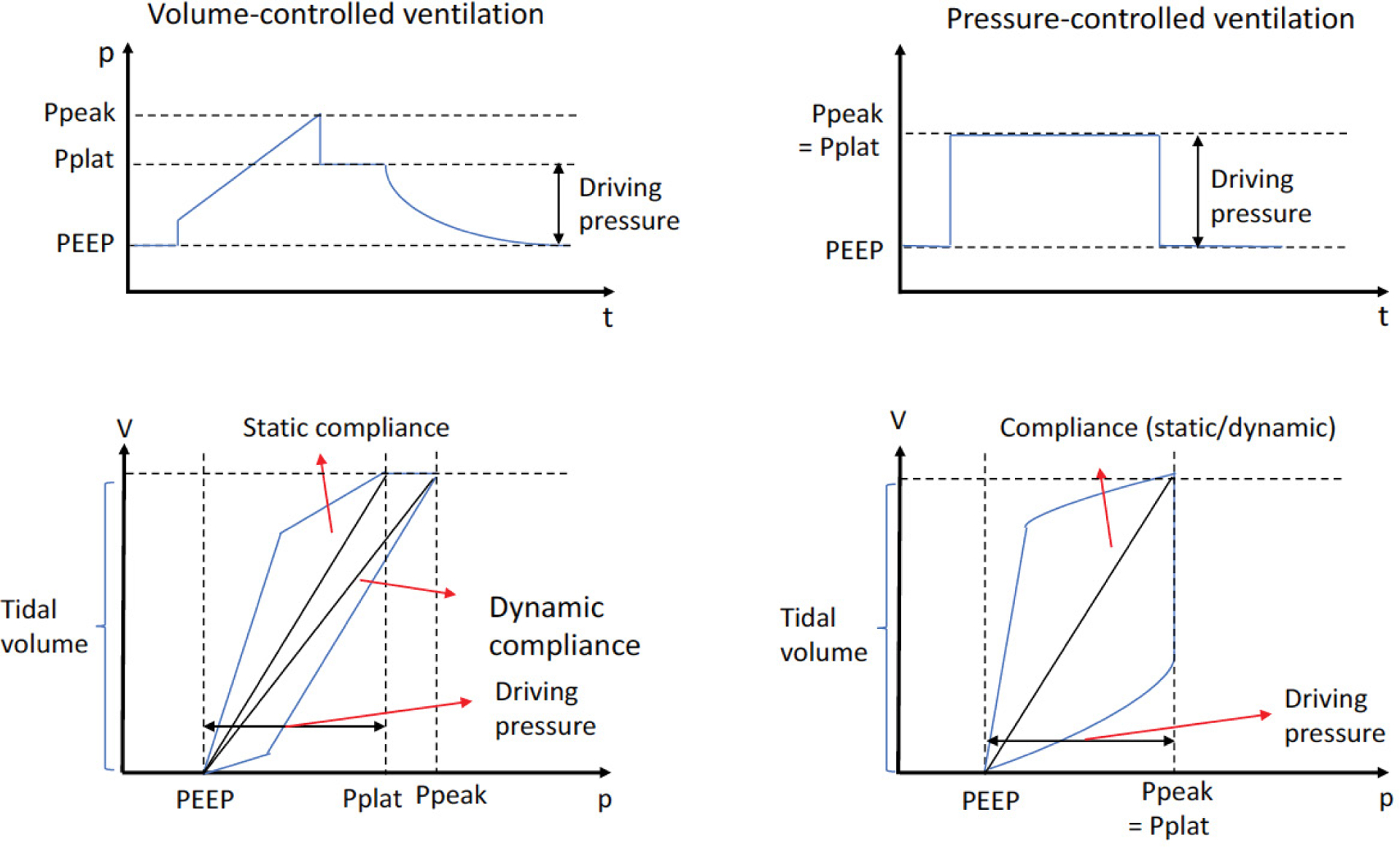
The relationship between pressure and time, and between pressure and volume. Ppeak: Peak Pressure; Pplat: Plateau Pressure; PEEP: Positive End-Expiratory Pressure

From early days of MV use, structural damage to the lungs by MV was well recognized [17]. This includes pneumothorax, pneumomediastinum, and subcutaneous emphysema [18]. Although the term VILI was only introduced in 1993 [19], various lines of preclinical investigation were done which proposed that excessive pressure (“barotrauma”) [20], excessive volume (“volutrauma”) [21] and the cyclic opening-closing of the lung units subjected to atelectasis (“atelectrauma”) [22] and biotrauma were considered four main causes for VILI as described in the following section. Subsequently, a number of clinical studies have been designed to test these concepts. Traditional MV regimen for Acute Respiratory Distress Syndrome (ARDS) was to use TV of 10–15 mL/kg [23]. The ARDS Network trial (ARMA trial) examined the outcome of patients with ARDS receiving lower and higher TVs to determine if higher TV would cause stretch-induced lung injury. The study demonstrated that the lower TV arm (6 mL/kg, mean plateau pressure 25 cmH_2_O) was associated with less mortality compared to the higher TV arm (12 mL/kg, mean plateau pressure 33 cm H_2_O) (mortality 31.0% vs. 39.8%) [23], supporting the idea that the ventilatory method could influence the outcome of patients. This result was in line with the concept of volutrauma and barotrauma. High positive end-expiratory pressure (PEEP) may also attenuate atelectrauma. Furthermore, IL-6 levels were higher in patients with mortality, supporting the concept of biotrauma in this patient cohort. Following this study, “lung protective” MV strategy using TV of approximately 6 mL/kg has been a common approach in patients with ARDS. Since the same TV can be attained under different pressures to the lungs, the role of pressure load posed to the lungs by MV in outcomes was also studied. Driving pressure is defined as a plateau pressure minus positive end-expiratory pressure (PEEP) (Pdriving = Pplat – PEEP) (Figure 1). Plateau pressure is measured at the end of an inspiratory pause during volume-controlled ventilation and at the end of inspiration during pressure-controlled ventilation [24]. An inspiratory pause greater than or equal to 3 seconds predicts plateau pressure with the best accuracy [25]. In contrast, an inspiratory pause of 0.5 second overestimates plateau pressure by 11% in ARDS patients [25]. At a fixed tidal volume, changes in driving pressure that occur as PEEP increases or decreases, reflect changes in respiratory system compliance. At the PEEP associated with the lowest driving pressure, respiratory system compliance is the highest. So PEEP can be titrated to attain this goal. Because driving pressure consists of the pressure distributed to the lungs themselves (transpulmonary pressure) and the pressure applied to the chest wall, it is not fully representative of the stress forced to the lungs. Nonetheless, in the study by Amato, et al., higher driving pressure was associated with an increased mortality in patients with ARDS, while PEEP or plateau pressure did not show any association with the mortality [26]. Relative risk of in-hospital death was 1.0 at the driving pressure of around 15 cmH_2_O. The role of driving pressure in outcomes was also examined by Fuller, et al. in patients who required MV but did not have ARDS at the initiation of MV. The mortality and ARDS development were used as outcome measures. Non-survivors were significantly associated with higher driving pressures (15.9 vs. 14.9 cmH_2_O) and higher plateau pressures (21.4 vs. 20.4 cmH_2_O) than survivors [27]. In theory, a transpulmonary pressure should be taken into consideration rather than the driving pressure alone due to the reason described above. Nevertheless, these studies demonstrated that the driving pressure was an important parameter to consider for MV. On average, the ratio of lung elastance to total respiratory system (lung and chest wall) is 0.7. If the driving pressure is 20 cmH_2_O, transpulmonary pressure is 14 cmH_2_O. But the ratio can range from 0.2 to 0.8 [28]. The transpulmonary pressure is the same 14 cmH_2_O when the driving pressure is 28 cmH_2_O and the ratio is 0.5. Transpulmonary pressure is the pressure difference between the alveoli and the esophagus, requiring an esophageal pressure probe. The use of esophageal probe was shown to significantly improve oxygenation and compliance during MV in ARDS patients [29], though this measurement is not a routine in the clinical practice. Overall, a number of clinical studies in ICU patients has indicated that MV strategies could have an impact on patient outcomes.

### Ventilator-induced lung injury in the operating room settings

MV is also utilized for general anesthesia during surgical procedures. In the operating room setting, patients who may have normal lung function prior to surgery receive MV for a short period [30]. Postoperative respiratory complications represent the second most common perioperative complication after wound infection, with an estimated incidence of 3.0–10.0% [31–34]. Because the majority of patients requiring MV in the ICU settings has pre-existing lung injury, the deterioration of respiratory status following MV may not be necessarily attributed to MV. In this sense, surgical patients may be more homogeneous from pre-MV lung wellbeing and easier in order to understand VILI.

A number of studies have examined the association between MV settings and postoperative complications. Futier, et al. performed the Intraoperative Protective Ventilation (IMPROVE) prospective trial to determine whether protective ventilation could improve outcomes after elective abdominal surgery [35]. In the study, patients aged 40 years and older received volume-controlled MV either with TV of 10–12 mL/kg, no PEEP, no recruitment maneuvers or with TV of 6–8 mL/kg, PEEP of 6–8 cmH_2_O and recruitment maneuvers every 30 minutes (lung protective ventilation). The primary outcome was a composite of major pulmonary (pneumonia, postoperative need for invasive or noninvasive ventilation) and extrapulmonary (sepsis, death) complications by postoperative day 7. Lung protective ventilation was associated with significantly less postoperative composite complications. Severgnini, et al. also prospectively examined the role of lung protective ventilation in open abdominal surgery [36]. In this study, patients aged 18 years and older received volume-controlled ventilation either with TV of 9 mL/kg, no PEEP or TV of 7 mL/kg, PEEP of 10 cmH_2_O and recruitment maneuvers (lung protective). Lung protective ventilation was associated with higher modified Clinical Pulmonary Infection Score (mCPIS) and better oxygenation on postoperative day 1 and 3. These studies paired high PEEP with low TV. To understand the role of PEEP in low TV ventilation, the Protective Ventilation using High versus Low PEEP (PROVHILO) trial was performed in open abdominal surgery [37]. Patients aged 18 years and older were enrolled for ventilation with TV of 8 mL/kg, PEEP of 12 cmH_2_O and recruitment maneuver or the same TV and PEEP < 2 cmH_2_O. The primary outcome was a composite of postoperative pulmonary complications (hypoxemia, bronchospasm, suspected pulmonary infection, pulmonary infiltrate, aspiration pneumonitis, ARDS, pleural effusion, pulmonary edema, pneumothorax) within the first 5 days after surgery. No difference in the incidence of postoperative pulmonary complications was observed between the two groups. However, this study did not clarify if PEEP level between 2 and 12 cmH_2_O had any role or not. In addition, it is important to point out that PROVHILO trial did not include laparoscopic procedures or morbidly obese patients, different from IMPROVE trial. Realizing these differences, these studies demonstrated the importance of low TV ventilation in patients undergoing abdominal surgery. However, a recent study of examining optimal tidal volume by Karalapillai, et al. added more complexity into this topic. In the study, they randomized patients to TV of 6 mL/kg versus 10 mL/kg with PEEP of 5 cmH_2_O in major noncardiothoracic, non-intracranial surgery under general anesthesia [38]. The two groups did not show any major difference in pulmonary complications. However, when they did subgroup analysis of abdominal surgery cohorts, there was a trend of less complications in the lower TV group, although statistically not significant. Thus, the benefit of a certain ventilation regimen may be considered based on the type of procedures at least from the outcome standpoint. In this line, de Jong, et al. analyzed the correlation between intraoperative PEEP levels and postoperative pulmonary complications (respiratory failure, reintubation, pneumonia, pulmonary edema) in patients aged 18 years and older undergoing abdominal surgery and craniotomies [34]. Application of PEEP of > 5 cmH_2_O was associated with significantly less respiratory complications in patients undergoing abdominal surgery, but not in patients undergoing craniotomies. During abdominal surgery, a number of surgical maneuvers often contribute to a cephalad displacement of the diaphragm, which authors postulated as an explanation for the difference. Of note, TV was not evaluated in this study. Ladha, et al. incorporated a variety of non-cardiac surgeries and evaluated the association between the composites of postoperative respiratory complications (pneumonia, respiratory failure, pulmonary edema, re-intubation) and protective MV in 69,265 patients [30]. Protective ventilation was defined as median PEEP of 5 cmH_2_O or greater, a median expiratory TV of < 10 mL/kg predicted body weight, and a median plateau pressure of < 30 cmH_2_O. The study demonstrated that PEEP of 5 cmH_2_O and a plateau pressure of < 16 cmH_2_O were identified as lung-protective. TVs did not show any correlation with a composite of respiratory complications, but the majority of the patients were ventilated with TV of < 10 mL/kg.

Serpa Neto, et al. performed meta-analysis of the data from randomized controlled trials to evaluate the role of protective ventilation under general anesthesia during surgery with the primary outcome to be the occurrence of postoperative pulmonary complications (postoperative lung injury, pulmonary infection) [39]. Higher driving pressure was associated with the development of postoperative pulmonary complications, but neither TV nor PEEP showed any association. Importantly, an increase in the level of PEEP that resulted in an increase in driving pressure was associated with more postoperative pulmonary complications. In other words, when an increase in PEEP does not contribute to an improvement of respiratory compliance, the PEEP level can be injurious by overstretching the aerated parts of the lung.

Overall, these studies pointed out the importance of selection of optimal TV, driving pressure, plateau pressure and PEEP in the perioperative setting where patients tend to have normal lung function prior to MV as in MV.

### Study design of VILI using rodents

A preclinical model is an important tool to test different ventilatory methods under controlled conditions and determine the mechanisms of VILI with the luxury of tissue sampling. A number of investigators have used different models to dissect these underlying mechanisms. Barotrauma, volutrauma, atelectrauma and biotrauma are four classic mechanisms of VILI development that have been described so far [40]. In regards to the different animal models, overall, the major difference is in the species used. Specifically, the use of large animals is effective to explore the effects of gravity on the development of VILI [6], but it is expensive and requires a large facility to accommodate. On the other hand, rodents such as mice, are much more affordable, although measuring their lung function may be more challenging than large animals [7]. However, TV, pressure and flow of rodents can be measured with commercially available equipment now. CT scan for rodents is also available for volume measurement. Here we will focus on studies describing how mice have been ventilated for mechanistic studies.

One of the major indications to use MV in mice is to provide procedural anesthesia for a short duration. For this purpose, most of the experiments have used respiratory rate (RR) of 100 to 150/min, TV of 200–700 μL (4–20 mL/kg) and inspiratory to total cycle duration ratio (Ti/Tt) of 0.2–0.4. To study VILI with a range of TVs, the majority of mouse studies have also used short-term mechanical ventilation (Table 2 and Table 3). VILI studies are divided into one-hit (Table 2) and two-hit (Table 3) models. Two-hit models have been usually done by lipopolysaccharide (LPS) instillation or cecal ligation and puncture (CLP) surgery followed by MV. The choice between one hit and two hit models should be made based on experimental paradigm. One hit model is advantageous to directly examine lung injury by MV only, but pure clinical scenario mimicking this may be restricted to perioperative MV for surgical anesthesia. Two hit model is more realistic scenario for MV use in ICU setting. LPS has been one of the major hits used for two hit models. LPS is not necessarily an infection model, so the relevance of this model should be considered in a case by case manner. In addition, mouse is more resistant to LPS than human. As described above, interspecies differences in professional phagocyte responses, particularly in the setting of the first hit, induced by infection may be important to consider when using the two hit model. CLP model has been also used as the initial hit. In this model, a large number of neutrophils accumulate after CLP, which may lower the threshold of VILI. Although extra-pulmonary infection is one of major causes for MV use, pneumonia is another indication. Using pulmonary infection model for MV should be also considered if that fits for experimental paradigm.

Wilson, et al. described that TV of > 30 mL/kg (PEEP 3 cmH_2_O, RR 80/min) was necessary to induce VILI by barotrauma/volutrauma in mice within 3 hours [41], while ventilation with a smaller TV and no PEEP may be useful for studying atelectrauma [42]. Long durations of MV have been also studied in mice. Szabari, et al. described mouse MV for up to 16 hours [42]. They ventilated mice with low TV of 6 mL/kg (PEEP 2 cmH_2_O, RR 180/min) or moderate TV-15 ml/kg (PEEP 2 cmH_2_O, RR 80/min) or 20 mL/kg (PEEP 2 cmH_2_O, RR 52/min)- to explore whether injury induced by alveolar decruitment or stretch would be reflected by differing mechanical responses, inflammation, and lethality. They then investigated differences between survivors and non-survivors. Although TV of the 6 mL/kg group had better survival, this group also developed lung injury, certainly indicating the importance of the duration of MV. In fact, higher IL-6 levels were associated with mortality irrelevant of ventilatory maneuvers, which is also in line with biotrauma theory. To further support the role of biotrauma in VILI, Bertok, et al. used selective inhibition of p55 tumor necrosis factor (TNF) receptor while ventilating mice with TV of 22 mL/kg [43]. TNF receptor inhibition attenuated lung injury, supporting the role of biotrauma in VILI. Li, et al. examined the role of signaling pathway in VILI development [16]. Injurious lung ventilation induced WNT1 expression, which contributed to TLR4 signaling activation. The role between WNT1 and TNF receptor signaling is not clear yet, but the data by Li, et al. strongly support biotrauma-induced VILI. As showed in the data by Li, et al. [16], the selection of mouse strains could have significant effect on VILI phenotypes, which were at least in part attributed to the difference in the signaling pathway activation. A/J mice were more vulnerable to VILI, while Balb/c were most resistant, with C57/BL6 to be in between the two strains. This intra-species difference was associated and in accordance with the difference in WNT1-TLR4 signaling pathway activation observed between the different species.

As described above, various TVs have been used for VILI studies. How should we choose TV? During tidal breathing, the change in lung volume is TV, and the initial lung volume corresponds to the functional residual capacity (FRC). Global volumetric lung strain can be estimated as TV/FRC. FRC in ARDS patients can be very small, which signifies the importance of low TV (6 mL/kg) management, whereas patients with healthy lung in the operating room setting may have high FRC, and TV at the range of 10 mL/kg may not contribute to the strain. This may be in line with the meta-analysis study of surgical patients that did not show any role in TV [39]. FRC of healthy mice breathing spontaneously was previously determined using CT scan [44]. FRC increased between 4–6 weeks-old and then remained stable after 6 weeks of age with a range of 200–400 μL for C3H and A/J mice. Titration of TV should be considered to mimic strain intended to pose to the mouse lung in reference to FRC. For example, TV of 10 mL/kg for 30 gm mouse (i.e. 300 μL) increases volume by two times if FRC is 300 μL. Baseline FRC measurement in an individual ventilatory condition using micro-CT in mice, would significantly enhance our understanding about the degree of strain contribution. For example, Yen, et al. performed ventilation in Balb/C mice either with PIP of 12 cmH_2_O, PEEP of 2 cmH_2_O or with PIP of 20 cmH_2_O, PEEP of 0 cmH_2_O [45]. FRC and TV for the former setting were 700–800 μL and 150–200 μL, respectively, while FRC and TV for the latter setting were 600–750 μL and 200–500 μL. Some PEEP or recruitment breath should be included to avoid atelectasis unless atelectrauma is the major focus of research. It is critical to understand the effect of PEEP on FRC at the same time.

### Future rodent studies

Clinical studies have been often limited to examining the correlation between ventilatory settings and postoperative outcomes, without the assessment of lung parenchyma. Ventilation heterogeneity is widely considered as a marker of pulmonary dysfunction in ARDS and VILI [46]. The heterogeneous change of lung parenchyma results in a maldistribution of ventilation. More compliant regions of the lung are prone to overventilation and over-distension. Stiffer regions of the lung are prone to under-ventilation and at risk of decruitment [47]. These mechanical burdens are associated with injurious, inflammatory responses in the lung. The association between ventilation heterogeneity and VILI is well illustrated in a number of studies of supine versus prone ventilation. Compared to ventilation in a supine position, being in a prone position improved homogeneity within the lung [48]. In patients with severe ARDS, patients ventilated in prone position showed significantly lower mortality than in supine position [49].

It is intuitive that MV can affect the already heterogeneous lung, thereby further aggravating this heterogeneity. However, does MV itself contribute to this heterogeneity in the healthy lung? It is well described that induction of general anesthesia promotes a reduction in lung volume and atelectasis formation associated with a deterioration of both gas exchange and respiratory mechanics [50,51]. Does the transition from spontaneous ventilation to positive pressure ventilation contribute to this? One of the important characteristics of spontaneous ventilation is the motion of the diaphragm. Diaphragm is contracted during expiration, thus preserving distal airway patency and avoiding/reducing expiratory atelectasis formation [52]. In addition, the dorsal and more compliant part of the diaphragm moves preferentially during spontaneous breathing, which helps to improve ventilation-perfusion matching [53]. During positive pressure ventilation in the supine position, however, the diameter of the distal airway in the dorsal part of the lung, which carries more volume than the ventral part, can become significantly narrower, leading to airway collapse. Thus, positive pressure MV itself in the setting of limited active diaphragm function can contribute to the development of lung heterogeneity, which may explain the association between ventilation settings and postoperative complications in the OR setting. An adequate level of PEEP could circumvent the airway collapse on the dependent region of the lung, while minimizing overstretches the more compliant part of the lung. Parameters such as driving pressure, PEEP and TV are output in the lung as a whole, thus, understanding the impact of these parameters on different regions of the lung should be one of the important directions in VILI research.

TV, driving pressure and plateau pressure are parameters that are measured at a static state during respiratory cycle [54]. However, respiratory rate (RR) should be also an important parameter. Flow to airway before air reaches the lung alveoli requires energy and poses stress to the airway. PEEP poses stress to the lungs at the baseline. Mechanical power is a concept developed based on the hypothesis that the degree of VILI depends on the amount of energy transferred from the ventilator to the lungs [55]. Mechanical power is determined by TV, plateau pressure, PEEP, respiratory rate and air flow and is expressed as the amount of energy per minute. A study of ICU patients requiring MV showed that higher mechanical power was associated with increased ICU mortality, in-hospital mortality and 30-day mortality [56]. Another study of ICU patients also showed that higher mechanical power was associated with increased mortality [27]. These studies supported the importance of the concept of mechanical power. Respiratory rate that has been used in the studies so far ranges from 20/min to 160/min. Increased respiratory rate can increase plateau pressure, thereby increasing driving pressure [57]. Although the selection of respiratory rate is important to keep PaCO_2_ in a desired range, it needs to be chosen carefully given it could affect plateau pressure. The mechanistic analysis of the relationship between mechanical power and VILI needs to be examined extensively in the future. Pressure-controlled ventilation is likely associated with less mechanical power than volume-controlled ventilation given that resistance due to flow may be much less [58], but the data so far have not indicated any superiority of one ventilation mode over the other [59]. Overall, using optimal mechanical power (TV, plateau pressure, PEEP, RR) to achieve appropriate gas exchange without or minimizing a significant strain and stress to the focal susceptible lung should be the goal of MV. It is also important to keep in mind that the duration of MV is not taken into consideration in mechanical power. Furthermore, the duration of MV is another component to be considered extensively because the study by Szabari, et al. demonstrated the contribution of longer MV to VILI.

As the importance of MV-associated inflammatory responses in VILI pathophysiology has been demonstrated to many different studies [6] (Table 2 and Table 3), rodent experiment addressing this concept would allow us to fully study immunological responses and other molecular mechanisms contributing to VILI for future intervention.

## Conclusion

In conclusion, MV is an important medical tool but still needs sophistication to reduce complications in clinical management. Preclinical models are and will continue to be important tools to supplement the void of clinical studies, but step-by-step approaches and well-defined and different combination of parameters will be necessary for optimal translational potential in order to obtain the correct answers for our clinical questions.

## Figures and Tables

**Table 1:** Anatomical difference between mouse and human lungs.

	Mouse	Human
**TLC**	1 mL	5,000–6,000 mL
**Lobes**	4 right lobes, 1 left lobe	3 right lobes, 2 left lobes
**Parenchyma**	18% of total lung	12% of total lung
**Airway generation**	13–17 generations	17–21 generations
**Alveoli**	80 μm MLI	210 μm MLI
**Blood-gas barrier thickness**	0.32 μm	0.62 μm

**Table 2:** Rodent one-hit VILI model.

Species/Age (if available)	MV setting	Duration	Results	References
**Mouse**				**-**
	VT of 7.5–8 ml/kg PEEP of 2–4 cmH_2_O	4–6h	Higher inflammatory cytokines in serum/BALF, recruitment of pulmonary granulocytes, moderate lung edema, and increased permeability of the alveolar–capillary barrier	Vaneker et al. [60]; Reiss, et al. [61]
	TV of 7.5 ml/kg or 15 ml/kg PEEP of 2 cmH_2_O FiO_2_ = 0.5	5h	Minor lung histopathological changes. MV in both settings caused higher wet-to-dry ratios, higher BALF protein levels and more influx of neutrophils, higher levels of proinflammatory cytokines and coagulation factors and higher systemic levels of cytokines. All parameters were higher in the larger TV group.	Wolthuis, et al. [62]
	High TV of 34.5 +/− 2.9 ml/kg Low TV of 8.8 +/− 0.5 ml/kg	2–3h	High TV: Progressive lung injury with a decrease in respiratory system compliance, increase in protein concentration in BALF, and lung pathology showing hyaline membrane formation. Increased MIP-2 in BALF. Low TV: Minimal changes in physiology and pathology with negligible TNF-alpha and MIP-2 proteins	Wilson, et al. [63]
	High TV of ^~^15 mL/kg, PEEP of 0 cmH_2_O, RR 52/min. FiO_2_ of 0.5 and I:E ratio of 1:3 Low TV of ^~^7 mL/kg, PEEP of 3 cmH_2_O RR of 160/min	5–12h	In both MV groups, PaO_2_/FiO_2_ ratios were lower and alveolar cell counts were higher after 12 hours of MV compared to 5 hours. Alveolar-capillary permeability was increased after 12 hours compared to 5 hours. Only in mice ventilated with higher TV, lung compliance declined and wet to dry ratio increased after 12 hours of MV compared to 5 hours.	Hegeman, et al. [64]
6–8 weeks-old.	High-peak pressure/stretch protocol: Peak pressure of 40 cm H_2_O (TV of ^~^24 ml/kg), RR of 100/min, FiO_2_ of 0.21) Low-peak pressure/stretch protocol: Peak pressure of 20 cm H_2_O (TV of 12 mL/kg, RR of 100/min, FiO_2_ of 0.21)	6h	Lung injury and neutrophil sequestration from the high-peak pressure/stretch group were greater than those from the low-peak pressure/stretch group. Lung expression of KC/CXCL1 and MIP-2/CXCL2/3 paralleled lung injury and neutrophil sequestration. *In vivo* inhibition of CXCR2/CXC chemokine ligand interactions led to a marked reduction in neutrophil sequestration and lung injury	Belpeiro, et al. [65]
8–12 weeks-old	High TV: TV of 28 ml/kg, PEEP of 0 cmH_2_O, RR of 60/min. Normal TV: TV of 7 ml/kg, PEEP of 0, RR of 120/min	4h	MV with higher TV activated the NLRP3 inflammasomes in mouse alveolar macrophages and increased the production of IL-1β *in vivo*. IL-1β neutralization significantly reduced MV-induced inflammatory lung injury	Wu, et al. [66]
**Rat**				
	TV of 10 ml/kg	2h	Increased expression of proinflammatory cytokines in macrophages of BALF	Kotani, et al. [67]
	TV of 8 ml/kg, PEEP of 0 cmH_2_O	4h	Disruption of the extracellular matrix, with perivascular space engorgement, cuff formation, and substantial alterations of lung mechanics	Moriondo, et al. [68]
	PIP of 32 cmH_2_O, PEEP of 0 cmH_2_O (ZEEP) PIP of 32 cmH_2_O, PEEP of 6 cmH_2_O PIP of 14 cmH_2_O, PEEP of 6 cmH_2_O For 1–3, FiO2 of 1.0, I:E ratio of 1: 1:2, RR of 20–30/min PIP of 32 cmH_2_O, PEEP of 6 cmH2O. FiO2 of 0.15–1.0	4h	All immune measurements in the low PIP/PEEP group did not differ from the immune measurements in the reference group. High PIP strategies, irrespective of applied PEEP, enhanced MIP-2 levels in lung and plasma. NK cell activity, mitogen-induced splenocyte proliferation and MIP-2 and IL-10 production significantly decreased after high PIP/PEEP ventilation. In the high PIP/ZEEP-ventilated group, the decrease in splenocyte proliferation, MIP-2 and IL-10 production and NK cell activity was more pronounced and interferon-γ production was also significantly lower than in the low PIP/PEEP group.	Vreugdenhil, et al. [69]

**Table 3:** Rodent two-hit VILI model.

Species/Age (if available)	MV setting	Duration	Results	References
**Mouse**				-
8–10 weeks-old	TV of 10 mL/kg, PEEP of 0 cmH_2_O, RR of 150/min, and an FiO_2_ of 0.21 +/− LPS	4–6 h	MV resulted in no significant pulmonary inflammation or injury and only modest differential gene expression compared with non-ventilated controls. When MV was combined with LPS, there was broad augmentation of gene transcription, which was associated with enhanced inflammation and the development of lung injury.	Altemeier, et al. [70]
8–10 weeks	Spontaneous breathing Spontaneous breathing with CLP MV: TV of 10 ml/kg, PEEP of 0 cmH_2_O, RR of 150/min CLP and MV	6h	MV itself did not cause lung injury. It exacerbated increases in alveolar-capillary permeability, histopathologic scoring and indices of pulmonary inflammation in mice that underwent CLP. The effects of this two-hit model were abrogated in TLR4−/− mice. Attendant with these findings was a significant increase in intrapulmonary WISP1 and integrin β5 in the two-hit model. Anti-WISP1 or antiintegrin β5 antibodies partially inhibited the two-hit phenotype. In peritoneal macrophages (PM), activation of TLR4 led to an increase in integrin β5 expression that was MyD88 and NF-κB dependent. Recombinant WISP1 increased LPS-induced cytokine release in PM (TNF-α, IL-6, MIP-2, MCP-1) that was inhibited by silencing either TLR4 or integrin β5.	Ding, et al. [71]
	Spontaneous breathing LPS + spontaneous breathing Low tidal volume (LV_T_) ventilation High tidal volume (HV_T_) ventilation LPS + HV_T_ LPS + LV_T_ HV_T_: RR of 45 breaths/min; TV of 20 ml/kg; and PEEP of 0 cm H_2_O (high tidal volume, HV_T_ group). LV_T_: RR of 135 breaths/min; TV of 10 ml/kg; and PEEP, 2 cmH_2_O	4h	Although LPS recruited neutrophils to airways, the addition of HV_T_ was required for significant induction of NETs markers. HV_T_ increased airway HMGB1 protein and IL-1β in LPStreated mice and tended to increase MCP-1 IL-6. Intratracheal DNase treatment reduced NET markers and attenuated the loss of static compliance without significantly impacting other measures of injury. Blockade of HMGB1 (with glycyrrhizin) or IL-1β (with anakinra) did not prevent NETosis or protect against injury.	Yildiz, et al. [72]
**Rat**				
	Spontaneous breathing Spontaneous breathing with LPS MV MV with LPS MV: TV of 10 ml/kg, PEEP of 0 cmH_2_O, RR of 40/min), FiO_2_ = 0.21	4h	MV significantly augmented LPS-induced lung injury and HMGB1 expression, which was correlated with the increase in IL-1β, IL-6 and MIP-2 levels in BALF.	Ding, et al. [73]
	LPS + 16 cmH_2_O PIP and 5 cmH_2_O PEEP LPS + 26 cmH_2_O PIP and 5 cmH_2_O PEEP LPS + 35 cmH_2_O PIP and 5 cmH_2_O PEEP	3h	MV creates an alveolar/pulmonary antifibrinolytic milieu in LPS-induced lung injury which, at least in part, might be due to an increase in plasminogen activator inhibitor activity. Specifically, LPS-induced lung injury increased TATc, D-dimer and PAI activity and PAI-1 antigen levels *versus* healthy animals. High pressure-amplitude ventilation increased TATc concentrations. D-dimer concentrations were not significantly raised. Instead, PAI activity increased with the amplitude of the pressure, from 0.7 U·mL^−1^ in group 1 to 3.4 U·mL^−1^ in group 2 and 5.0 U·mL^−1^ in group 3. There was no change in PAI-1 antigen levels.	Dahlem, et al. [74]
	Low tidal volumes (LTV) LTV with LPS High tidal volumes (HTV) HTV with LPS. HTV: 19 ml/kg, PEEP at 1 cmH_2_O and RR of 20/min LTV: 6 ml/kg, PEEP at 5 cmH_2_O, RR of 45/min	4h	LTV ameliorated LV systolic and diastolic dysfunction while preventing death following LPS-induced lung injury in mechanically ventilated rats. Specifically, Ees/Ea decreased over time in rats receiving LPS and HTV, with a lower Ees/Ea in the rats with HTV plus LPS compared to the other groups. Eed increased over time in all groups except for the rats receiving LTV without LPS. A significant interaction was found between TV and LPS for Ees/Ea and Eed, and all rats receiving HTV plus LPS died before the end of the experiment.	Cherpanath, et al. [75]
